# Supplementary material for: Folding of cohesin’s coiled coil is important for Scc2/4-induced association with chromosomes
Source: eLife. 2021 Jul 14;10:e67268. doi: 10.7554/eLife.67268 (PMC8279761; doi:10.7554/eLife.67268)
Supplement: Supplementary file 1. [file elife-67268-supp1.docx]

Supplementary File 1

| Residue at 588 | Complements *smc1∆*? | Complements *scc4∆*? |
| --- | --- | --- |
| Asp (D) | YES | NO |
| Tyr (Y)  Phe (F)  Trp (W) | YES  YES  YES | YES  YES  YES |
| His (H)  Ala (A)  Glu (E)  Arg (R)  Asn (N) | YES  YES  YES  YES  YES | NO  NO  NO  NO  NO |
