# Supplementary material for: Folding of cohesin’s coiled coil is important for Scc2/4-induced association with chromosomes
Source: eLife. 2021 Jul 14;10:e67268. doi: 10.7554/eLife.67268 (PMC8279761; doi:10.7554/eLife.67268)
Supplement: Supplementary file 2. [file elife-67268-supp2.docx]

**Supplementary File 2** Data collection and structure refinement statistics

| Data Collection | mmSmc1(471-685)D574Y-mmSmc3(484-696) |
| --- | --- |
| Space group | *P*1 |
| Unit cell dimensions |  |
| a, b, c (Å) | 50.08, 60.85, 77.76 |
| α, β, γ (°) | 72.66, 88.43, 89.99 |
| Wavelength (Å) | 0.97960 |
| Resolution (Å) | 42.06-2.00  (2.03-2.00)^a^ |
| R_sym_ (%) | 6.4 (17.6) |
| I/σ(I), (> 0) | 12.2 (2.7) |
| Completeness (%)^b^ | 88.6 (76.6) |
| Redundancy | 2.6 (1.9) |
| Refinement |  |
| Resolution (Å) | 42.06-2.00 |
| No. of reflections | 52564 |
| R_work_ / R_free_ (%) | 21.9/26.9 |
| R.m.s deviations |  |
| Bond lengths (Å) | 0.007 |
| Bond angles (º) | 0.859 |
| Average B-values (Å^2^) | 20.64 |
| Ramachandran plot (%) |  |
| Favored | 97.7 |
| Allowed | 2.2 |
| Outliers | 0.1 |
| PDB ID | 7DG5 |

^a^The values in parentheses are statistics from the highest resolution shell.

^b^The values are for reflections with I/σ(I) > 0.
