# Supplementary material for: Folding of cohesin’s coiled coil is important for Scc2/4-induced association with chromosomes
Source: eLife. 2021 Jul 14;10:e67268. doi: 10.7554/eLife.67268 (PMC8279761; doi:10.7554/eLife.67268)
Supplement: Supplementary file 3. [file elife-67268-supp3.docx]

**Supplementary File 3. Cryo-EM data & model building statistics**

|  | Cohesin Scc2 structure |
| --- | --- |
| Data collection and processing |  |
| Voltage (kV) | 300 |
| Electron exposure (e^–^/A^2^) | 40-45 |
| Defocus range (µm) | -0.5 to -0.9 |
| Pixel size (Å) | 1.16 |
| Symmetry imposed | C1 |
| Initial particle images (no.) | 450,000 |
| Final particle images (no.) | 32,491 |
| Map resolution (Å) | 16.7 (8.5) |
| FSC threshold | 0.5 (0.143) |
|  | **Coiled-coil elbow structure** |
| Data collection and processing |  |
| Voltage (kV) | 300 |
| Electron exposure (e^–^/A^2^) | 40-45 |
| Defocus range (µm) | -0.5 to -0.9 |
| Pixel size (Å) | 1.16 |
| Symmetry imposed | C1 |
| Initial particle images (no.) | 4.5 million |
| Final particle images (no.) | 63,892 |
| Map resolution (Å) | 5.5 |
| FSC threshold  Refinement  Initial model used (PDB)  Model resolution (Å)  FSC threshold  Model resolution range (Å)  Map sharpening B factor (Å^2^)  Model composition  Non-hydrogen atoms  Protein residues  *B* factors (Å^2^)  Protein  R.m.s. deviations  Bond lengths (Å)  Bond angles (°)  Validation  MolProbity score  Clashscore  Poor rotamers (%)  Ramachandran plot  Favored (%)  Allowed (%)  Disallowed (%) | 0.143  Ab initio, Homology model derived from 2WD5  5.5 Å  0.143  5.5-10  -200  7974  1396  61.27  0.006  0.599  1.46 (96^th^ Percentile)  8.02  0.00  97.9  2.1  0.00 |
|  | **ATP-engaged cohesin structure** |
| Data collection and processing |  |
| Voltage (kV) | 300 |
| Electron exposure (e^–^/A^2^) | 45 |
| Defocus range (µm) | -0.5 to -0.9 |
| Pixel size (Å) | 1.16 |
| Symmetry imposed | C1 |
| Initial particle images (no.) | 224,719 |
| Final particle images (no.) | 108,015 |
| Map resolution (Å) | 6 |
| FSC threshold | 0.143 |
|  | **Cohesin-Pds5 structure** |
| Data collection and processing |  |
| Voltage (kV) | 300 |
| Electron exposure (e^–^/A^2^) | 42 |
| Defocus range (µm) | -0.5 to -0.9 |
| Pixel size (Å) | 1.16 |
| Symmetry imposed | C1 |
| Initial particle images (no.) | 455,705 |
| Final particle images (no.) | 6,259 |
| Map resolution (Å) | 14.8 |
| FSC threshold | 0.143 |
