# Supplementary material for: Folding of cohesin’s coiled coil is important for Scc2/4-induced association with chromosomes
Source: eLife. 2021 Jul 14;10:e67268. doi: 10.7554/eLife.67268 (PMC8279761; doi:10.7554/eLife.67268)
Supplement: Supplementary file 4. [file elife-67268-supp4.docx]

###### Supplementary File 4 - List of strains.

| K699 | *MATa, W303 wildtype* |
| --- | --- |
| B1969 | *MATa, Scc1-PK9::KanMX, Scc2_6xHis_FLAG6::KANMX, pBH829 (smc1K620TAG-Myc9 in YEplac181), pBH61 (BPA crosslink, Trp1)* |
| B1976 | *MATa, Scc1-PK9::KanMX, Scc3_6xHis_FLAG6::KANMX, pBH829 (smc1K620TAG-Myc9 in YEplac181), pBH61 (BPA crosslink, Trp1)* |
| B1983 | *MATa, Scc1-PK9::KanMX, pBH829 (smc1K620TAG-Myc9 in YEplac181), pBH61 (BPA crosslink, Trp1)* |
| B2020 | *MATa, SMC3HA6::HIS3, Scc1-PK9::KanMX, pBH829 (smc1K620TAG-Myc9 in YEplac181), pBH61 (BPA crosslink, Trp1)* |
| B2072 | *MATa, Pds5_6xHis_FLAG6::KANMX, Scc1-PK9::NatMX, pBH829 (smc1K620TAG-Myc9 in YEplac181), pBH61 (BPA crosslink, Trp1)* |
| B2079 | *MATa, Scc4_6xHis_FLAG6::KANMX, Scc1-PK9::NatMX, pBH829 (smc1K620TAG-Myc9 in YEplac181), pBH61 (BPA crosslink, Trp1)* |
| B2143 | *MATa, Scc1-PK9::KanMX, Scc2TEV215_6xHis_FLAG6::KanMX, pBH61 (BPA crosslink, Trp1), pBH829 (smc1K620TAG-Myc9 in YEplac181)* |
| B2144 | *MATa, Scc1-PK9::KanMX, Scc2TEV471_6xHis_FLAG6::KanMX, pBH61 (BPA crosslink, Trp1), pBH829 (smc1K620TAG-Myc9 in YEplac181)* |
| B2145 | *MATa, Scc1-PK9::KanMX, Scc2TEV668_6xHis_FLAG6::KanMX, pBH61 (BPA crosslink, Trp1), pBH829 (smc1K620TAG-Myc9 in YEplac181)* |
| B2149 | *MATa, Scc2TEV843_6xHis_FLAG6::KanMX, Scc1-PK9::NatMX, pBH61 (BPA crosslink, Trp1), pBH829 (smc1K620TAG-Myc9 in YEplac181)* |
| B2212 | *MATa, Pds5_6xHis_FLAG6::KANMX, Scc1-PK9::NatMX, pBH909 (smc1D588Y_K620TAG-Myc9 in YEplac181), pBH61 (BPA crosslink, Trp1)* |
| B2213 | *MATa, Scc2_6xHis_FLAG6::KANMX, Scc1-PK9::NatMX, pBH909 (smc1D588Y_K620TAG-Myc9 in YEplac181), pBH61 (BPA crosslink, Trp1)* |
| B2214 | *MATa, scc4-4::NatMX, Pds5_6xHis_FLAG6::KANMX, Scc1-PK9::NatMX, pBH829 (smc1K620TAG-Myc9 in YEplac181), pBH61 (BPA crosslink, Trp1)* |
| B2215 | *MATa, scc4-4::NatMX, Pds5_6xHis_FLAG6::KANMX, Scc1-PK9::NatMX, pBH909 (smc1D588Y_K620TAG-Myc9 in YEplac181), pBH61 (BPA crosslink, Trp1)* |
| B2216 | *MATa, scc4-4::NatMX, Scc2_6xHis_FLAG6::KANMX, Scc1-PK9::NatMX, pBH829 (smc1K620TAG-Myc9 in YEplac181), pBH61 (BPA crosslink, Trp1)* |
| B2217 | *MATa, scc4-4::NatMX, Scc2_6xHis_FLAG6::KANMX, Scc1-PK9::NatMX, pBH909 (smc1D588Y_K620TAG-Myc9 in YEplac181), pBH61 (BPA crosslink, Trp1)* |
| B2298 | *MATa, Scc1-PK9::KanMX, scc2Δ::natMX4, lys2::Scc2T150TEV3_His6_Flag6/HyGMX, pBH909 (smc1D588Y_K620TAG-Myc9 in YEplac181), pBH61 (BPA crosslink, Trp1)* |
| B2735 | *MATa, Scc1-PK9::KanMX, scc2Δ::natMX4, Smc1K620C-myc3::KiTrp1, lys2Δ::Scc2A153C_HIS6_FLAG6* |
| B2736 | *MATa, Scc1-PK9::KanMX, scc2Δ::natMX4, Smc1K620C-myc3::KiTrp1, lys2Δ::Scc2N159C_HIS6_FLAG6* |
| B2737 | *MATa, Scc1-PK9::KanMX, scc2Δ::natMX4, Smc1K620C-myc3::KiTrp1, lys2Δ::Scc2N164C_HIS6_FLAG6* |
| B2738 | *MATa, Scc1-PK9::KanMX, scc2Δ::natMX4, Smc1K620C-myc3::KiTrp1, lys2Δ: Scc2T169C_HIS6_FLAG6* |
| B2739 | *MATa, Scc1-PK9::KanMX, scc2Δ::natMX4, Smc1K620C-myc3::KiTrp1, lys2Δ::Scc2T176C_HIS6_FLAG6* |
| B2740 | *MATa, Scc1-PK9::KanMX, scc2Δ::natMX4, Smc1K620C-myc3::KiTrp1, lys2Δ::Scc2N179C_HIS6_FLAG6* |
| B2741 | *MATa, Scc1-PK9::KanMX, scc2Δ::natMX4, Smc1K620C-myc3::KiTrp1, lys2Δ::Scc2Q183C_HIS6_FLAG6* |
| B2742 | *MATa, Scc1-PK9::KanMX, scc2Δ::natMX4, Smc1K620C-myc3::KiTrp1, lys2Δ::Scc2N186C_HIS6_FLAG6* |
| B2743 | *MATa, Scc1-PK9::KanMX, scc2Δ::natMX4, Smc1K620C-myc3::KiTrp1, lys2Δ::Scc2V195C_HIS6_FLAG6* |
| B2744 | *MATa, Scc1-PK9::KanMX, scc2Δ::natMX4, Smc1K620C-myc3::KiTrp1, lys2Δ::Scc2N200C_HIS6_FLAG6* |
| B2745 | *MATa, Scc1-PK9::KanMX, scc2Δ::natMX4, Smc1K620C-myc3::KiTrp1, lys2Δ::Scc2N206C_HIS6_FLAG6* |
| B2736 | *MATa, Scc1-PK9::KanMX, scc2Δ::natMX4, Smc1K620C-myc3::KiTrp1, lys2Δ::Scc2Q212C_HIS6_FLAG6* |
| B3082 | *MATa, Smc1-myc3::KItrp1, scc2Δ::natMX4, lys2Δ::Scc2N200C_HIS6_FLAG6, Scc1-PK9::KanMX* |
| B3107 | *MATa, Scc1-PK9::KanMX, Smc1K620C-myc3::KiTrp1, scc2Δ::natMX4, lys2Δ::Scc2N200C_HIS6_FLAG6* |
| B3114 | *MATa, Scc1-PK9::KanMX, scc2Δ::natMX4, lys2Δ:: Scc2_HIS6_FLAG6, Smc1K620C-myc3::KiTrp1* |
| B3116 | *MATa, Smc1-myc3::KItrp1, Scc1-PK9::KanMX, scc2Δ::natMX4, lys2Δ::Scc2_HIS6_FLAG6* |
| K5828 | *MATa, scc2-4* |
| K7564 | *MATα, SCC2-HA6::HIS3* |
| K8326 | *MATα, scc4Δ::HIS3, leu2::scc4-4::LEU2* |
| K8504 | *MATa, scc4Δ::HIS3, leu2::scc4-4::LEU2, SCC2-HA6::HIS3* |
| K19012 | *MATa, PDS5-PK6::KANMX6* |
| K19624 | *MATa, scc4Δ::HIS3, smc1(D588Y)::HphNT1, SCC1-PK6::KanMX4* |
| K19813 | *MATa, scc4Δ::HIS3, leu2::scc4-4::LEU2, smc1(D588Y)* |
| K20110 | *MATα, leu2::SCC4-myc18::LEU2, SCC2-HA6::HIS3* |
| K20111 | *MATα, leu2::scc4(Y40N)-myc18::LEU2, SCC2-HA6::HIS3* |
| K20112 | *MATα, leu2::scc4(Y40H)-myc18::LEU2, SCC2-HA6::HIS3* |
| K20113 | *MATα, leu2::scc4(Y40A)-myc18::LEU2, SCC2-HA6::HIS3* |
| K20350 | *MATα, scc4Δ::HIS3, leu2::SCC4-myc18::LEU2, SCC2-HA6::HIS3* |
| K20351 | *MATα, scc4Δ::HIS3, leu2::scc4(Y40N)-myc18::LEU2, SCC2-HA6::HIS3* |
| K20352 | *MATα, scc4Δ::HIS3, leu2::scc4(Y40H)-myc18::LEU2, SCC2-HA6::HIS3* |
| K20353 | *MATa, scc4Δ::HIS3, leu2::scc4(Y40A)-myc18::LEU2, SCC2-HA6::HIS3* |
| K21388 | *MATa, SCC2-PK6::KanMX6* |
| K21416 | *MATa, smc1(D588Y)::HphMX4* |
| K21973 | *MATa/α, SCC4/scc4Δ::HIS3, SMC1/smc1::KanMX4, trp1/trp1::SMC1::TRP1* |
| K21974 | *MATa/α, SCC4/scc4Δ::HIS3, SMC1/smc1::KanMX4, trp1/trp1::smc1(D588Y)::TRP1* |
| K21990 | *MATa/α, SCC4/scc4Δ::HIS3, SMC1/smc1::KanMX4, trp1/trp1::smc1(D588F)::TRP1* |
| K21995 | *MATa, scc2-4::NatMX4, smc1(D588Y)::HphMX4* |
| K21999 | *MATa, scc4Δ::HIS3, leu2::scc4-4::LEU2, smc1(D588Y)::HphNT1, SCC1-PK6* |
| K22001 | *MATa, scc4Δ::HIS3, leu2::scc4-4::LEU2, SCC1-PK6::KanMX4* |
| K22005 | *MATa, SCC1-PK6::KanMX4* |
| K22009 | *MATa, SCC1-PK6::KanMX4, smc1(D588Y)::HphNT1* |
| K22012 | *MATa/α, SCC4/scc4Δ::HIS3, SMC1/smc1::KanMX4, trp1/trp1::smc1(D588W)::TRP1* |
| K23967 | *MATa, scc4Δ::HIS3 YCplac33/scc4-4/NatMX4* |
| K23997 | *MATa, sth1-3(S806L/T881M), SCC1-PK6* |
| K24031 | *MATa, sth1-3(S806L/T881M), smc1(D588Y)::HphNT1, SCC1-PK6* |
| K24032 | *MATa, sth1-3* |
| K24562 | *MATa, trp1::smc3(E1155Q)-PK6::TRP1, ura3::SMC1-myc9::URA3* |
| K24564 | *MATa, trp1::smc3(E1155Q)::TRP1, ura3::smc1(D588Y)-myc9::URA3, scc4Δ::HIS3, smc1(D588Y)::HphNT1* |
| K24568 | *MATa, scc4Δ::HIS3, leu2::scc4-4::LEU2, hta1(R31I)::KanMX4, SCC1-PK6::KanMX4* |
| K24574 | *MATa, hta1(R31I)::KanMX4, SCC1-PK6::KanMX4* |
| K24678 | *MATa, scc4Δ::HIS3, smc1(D588Y)::HphNT1, SCC2-PK6::KanMX6* |
| K24680 | *MATa, smc1(D588Y)::HphNT1, SCC2-PK6::KanMX6* |
| K24689 | *MATa, trp1::smc3(E1155Q)PK6::TRP1, ura3::smc1(D588Y)myc9::URA3, smc1(D588Y)::HphNT1* |
| K25378 | *MATa, PDS5-PK6::KanMX6, smc1(D588Y)::HphMX4* |
| K27359 | *MATα, smc1(S161C)-HA::HIS3, smc3(K160C)-PK6::KanMX6* |
| K27751 | *MATa, SCC1-PK6::KanMX6, scc4Δ::HIS3, leu2::scc4-4::LEU2, ura::ADH1promoter-OsTIR1-MYC9::URA3, PDS5-AID::KanMX4* |
| K28401 | *MATa, SMC3-PK6::KanMX4, SMC1-HA6::HIS3* |
| K28546 | *MATa, smc1(R578)-HA6::HIS3, SMC3-PK6::KanMX4* |
| K28583 | *MATa, SMC1-HA6::HIS3, smc3(V933C)-PK6::KanMX4* |
| K28585 | *MATa, smc1(R578C)-HA6::HIS3, smc3(V933C)-PK6::KanMX4* |

*C. glabrata*

| K23308 | *MATa, SCC1-PK9::NatMX4* |
| --- | --- |
| K25532 | *MATa, SCC1-HA3::NatMX4* |
